# “Why Am I Even Here If I Can’t Save the Patients?”: The Frontline Healthcare Workers’ Experience of Burnout during COVID-19 Pandemic in Mthatha, South Africa

**DOI:** 10.3390/ijerph20085451

**Published:** 2023-04-10

**Authors:** Noluyolo Fathuse, Khumbulani W. Hlongwana, Themba G. Ginindza

**Affiliations:** 1 Discipline of Public Health Medicine, School of Nursing & Public Health, University of KwaZulu-Natal, Durban 4041, South Africa; 2 Cancer & Infectious Diseases Epidemiology Research Unit (CIDERU), College of Health Sciences, University of KwaZulu-Natal, Durban 4041, South Africa

**Keywords:** burnout, frontline healthcare workers, COVID-19, pandemic, Mthatha

## Abstract

Introduction: Globally, the high prevalence of burnout in healthcare workers (HCWs) is of the utmost concern. Burnout is a state of emotional exhaustion, depersonalization and a decreased sense of personal accomplishment. While the 2019 Coronavirus (COVID-19) exacerbated the burnout prevalence among HCWs, limited studies have explored this phenomenon using qualitative methodologies in the Eastern Cape Province and South Africa generally. This study explored how frontline healthcare workers experienced burnout during the COVID-19 pandemic in Mthatha Regional Hospital. Methods: Ten face-to-face in-depth interviews were conducted with non-specialized medical doctors and nurses who directly cared for COVID-19-infected patients during the pandemic in Mthatha Regional Hospital (MRH). In-depth interviews were digitally recorded and transcribed verbatim. Data were managed through NVIVO 12 software before being thematically analyzed using Colaizzi’s analysis method. Results: Four main themes emerged from the analysis. These themes were burnout manifestation (emotional strain, detachment and irritability, uncertainty-induced fear, and anxiety, physical exhaustion, yet, low job accomplishment, dread and professional responsibility), precursors of burnout (occupational exposure to high mortality, staff shortages, elongated high patient volume and workload, disease uncertainties and consistent feeling of grief), alleviating factors of burnout (time off work, psychologist intervention, periods of low infection rate and additional staff), and the last theme was every cloud has a silver lining (improved infection prevention and control (IPC) measures, learning to be more empathetic, the passion remains and confidence grows). Conclusion: The COVID-19 pandemic brought about a rapid change in the work environment of healthcare workers who are the backbone of efficient healthcare services, thereby rendering them vulnerable to increased burnout risks. This study provides strategic information for policymakers and managers on developing and strengthening welfare policies to promote and protect frontline health workers’ well-being and work functioning.

## 1. Introduction

Since the discovery of the concept by Freudernberger in 1974, burnout has gradually been recognized as a psychological phenomenon with personal, professional and organizational consequences, which affect both the quality of services rendered and personal well-being [1,2,3]. In his study, Freudernberger observed certain traits among overworked personnel in a free clinic in New York. What was apparent in these workers were physical and behavioral symptoms, including fatigue, exhaustion, recurrent headaches, insomnia, gastrointestinal problems, shortness of breath, anger, frustration, depersonalization, use of substances to induce sleep and depression. He concluded that what he had observed was “burnout” [2,4], a phenomenon that he attributed to emotionally demanding, exhausting and underpaying workplaces, requiring personal involvement and self-motivation [3,4]. Building on the foundation laid by Freudernberger, Maslach expanded the definition of burnout as a syndrome of psychological response to chronic emotional and interpersonal stressors on the job, consisting of three dimensions, namely emotional exhaustion, depersonalization and decreased personal accomplishment in people who consistently work with people and their problems [2,5,6]. To date, Maslach’s work has become an authority in the field of burnout [4]. Taking it a step further, the World Health Organization (WHO) has classified burnout as one of the factors influencing the health status of professionals who experience poorly managed chronic workplace stress [7].

Predominantly, burnout is assessed using Maslach’s Burnout Inventory (MBI), a questionnaire that measures the three dimensions of burnout [4,5,6]. Maslach and colleagues developed the (MBI) as a popular practical quantitative measurement tool for burnout across a diverse occupational spectrum [4,8].

Due to the nature of healthcare workers’ (HCWs) work, they are at increased risk of burnout [9], as shown by the global burnout prevalence among HCWs [10,11,12], which ranges between 18 and 82% and is highest among nurses and doctors [13,14,15]. In sub-Saharan Africa (SSA), the prevalence of burnout varies between 40 and 80% [11]. Burnout among HCWs is not a new problem, as the 2003 cross-sectional survey involving 132 district-level doctors from 27 facilities around the Cape Town Metropolitan Municipality in the Western Cape revealed that 73% of medical doctors had burnout [12,16]. In 2020, a survey conducted in KwaZulu-Natal, which followed the three dimensions of Maslach’s Burnout Inventory (MBI), revealed a burnout prevalence of 59% among medical doctors [17]. Lack of hospital resources, poor infrastructure, lack of clinical support from supervisors, poor working conditions, staff shortages, working overtime, high workload, lack of organizational support, inadequate salaries, dealing with crises at work [12,17,18] have all been reported as significant contributors to burnout. These challenges are pervasive among HCWs [19].

The 2019 coronavirus (COVID-19) pandemic created conducive conditions for burnout to thrive among HCWs, given substantive changes in their usual practice and the delivery of services to patients [18]. As of 18 July 2022, nearly four million (3,999,751) positive cases and over one hundred thousand (101,918) deaths had been reported in South Africa since the first case in March 2020 [20]. The sharp increase in COVID-19 morbidity and mortality put pressure on HCWs’ workload, who had to contend with the overwhelming ethically and emotionally wrenching decisions of allocating scarce resources, the risk of infection and infecting family members, and the trauma of losing loved ones and colleagues to the incurable virus. These factors have caused significant emotional exhaustion, physical exhaustion, anxiety, depression and powerlessness in HCWs’ ability to manage their patients, thereby rendering them vulnerable to burnout [8,9,21,22,23,24]. The current evidence suggests that COVID-19 frontline HCWs in designated COVID-19 wards were exposed to extraordinary stress [25,26,27].

Frontline HCWs’ health and safety are of the utmost importance, given their role in patient care, more so in such time as the COVID-19 pandemic [25]. Few studies conducted during the pandemic showed that HCWs directly caring for COVID-19-infected patients suffered a myriad of mental, emotional and physical challenges, including burnout, and these studies have, to some extent, been associated with gender, age, profession, marital status and level of experience [9,18,25]. Therefore, it is important to acquire an in-depth understanding of how HCWs in MRH have experienced burnout during the COVID-19 pandemic, with a view to guiding the development of interventional support strategies for future pandemics [24]. To the best of our knowledge, there are currently no studies exploring HCWs’ experiences of burnout in the Province of Eastern Cape using qualitative methods.

## 2. Materials and Methods

### 2.1. Study Design

A study using exploratory qualitative design was conducted to gain deeper insights into the manifestation of burnout in HCWs during COVID-19 from the participants’ perspectives.

### 2.2. Study Setting and Participants

The study was conducted in MRH, Oliver Reginald (OR) Tambo district in Eastern Cape, South Africa. The MRH has specialist departments, such as internal medicine, family medicine, obstetrics and gynecology, surgery, anesthesia and pediatrics. However, for this study, we recruited nurses and doctors who had been assigned to COVID-19 wards, as they were most likely to be severely affected by intimate and sustained exposure to caring for patients diagnosed with COVID-19. Notably, during the COVID-19 pandemic, specific areas were turned into COVID-19 specialized wards, as the rapid spread of the disease could not have been anticipated. Using purposive heterogeneous sampling, we recruited doctors and nurses who had been displaced from their original departments to COVID-19 wards. The heterogeneity nature of our sampling enabled us to ensure a good mix of participants in terms of occupation, experience, gender and age. Lunch breaks were used to conduct in-depth interviews, and this arrangement had been cleared by the facility management. The sample size was determined through data saturation, which was reached at ten in-depth interviews, a point where no new themes emerged from the in-depth interviews. The literature asserts that data saturation can be reached at ten in-depth interviews with information-rich participants [28,29,30].

### 2.3. Data Collection

Using the interview guide approach, between 23 March 2022 and 4 May 2022, we conducted in-depth face-to-face interviews in English with ten participants, at a time (lunch breaks) and place convenient for them. The interviews lasted an average of 26 min (14 to 49 min). All participants signed informed consent before being interviewed, and the interviews were tape recorded (with the participants’ permission). The in-depth interview explored how burnout manifested in those involved in caring for patients with COVID-19 (covering different elements of burnout), the effects of burnout on their health and patient care, as well as the effects of burnout on how they perceived their professions. To enhance the depth of the data, probing and practical questions, such as “*What had happened?*”; “*How did you handle the situation?*”, were also used. The interview was concluded by soliciting the participant’s socio-demographic information. For confidentiality purposes, participants were de-identified during the analysis.

### 2.4. Data Analysis

Data collection and analysis were performed iteratively. The audio recordings of the interviews were transcribed verbatim by the lead researcher and were then analyzed using Colaizzi’s method of analysis [31]. The process included reading the transcripts several times to understand and become familiar with the data, identifying relevant statements and making meaning of them, and organizing the data into categories, common themes and sub-themes [31] with the assistance of NVivo12 software [32]. The analysis was conducted inductively, thereby allowing themes to emerge from the data.

### 2.5. Measures to Ensure the Trustworthiness of the Study

To ensure the trustworthiness of the study, we followed Lincoln and Guba’s [33] guidance on credibility, transferability, dependability and confirmability. These were achieved through debriefing sessions after the initial interviews, providing a thick description of the study participants and the context, using verbatim quotes to support the findings and properly documenting the study processes. Since the lead researcher is a HCW herself and holds views on the subject matter, she constantly used bracketing to ensure that the study findings were free from her personal beliefs, values and opinions while maintaining rigor.

## 3. Results

**Figure 1:**
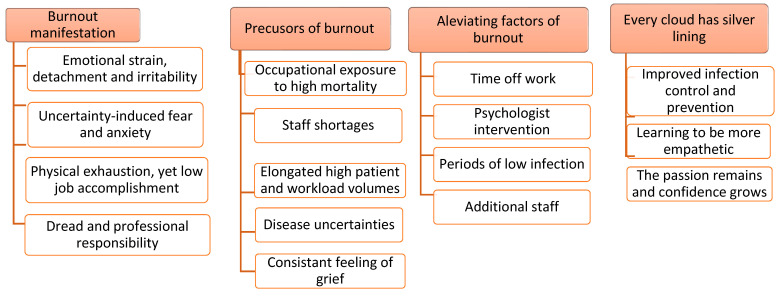
Themes and sub-themes from data analysis.

The study participants comprised six doctors and four nurses involved in caring for COVID-19-positive patients during the pandemic, with the ages ranging from 26 to 46 years and the majority (70%) being females. These participants were recruited from their original departments of work, including family medicine (three), internal medicine (three), gynecology (one), labor ward (one), general surgery (one) and high care (one). Eight and two participants were single and married, respectively. By designation, among the nurses, three were professional nurses and one was an enrolled nursing assistant compared to five medical officers and one family medicine registrar. The duration of working with confirmed COVID-19 patients ranged from 1 month to 24 months. Notably, the results of this study are analyzed without any emphasis on the specificities of the professionals interviewed (doctors vs. nurses). During data analysis, four main themes and sixteen sub-themes emerged from the analysis. These themes were burnout manifestation, precursors of burnout, alleviating factors of burnout, and lastly, every cloud has a silver lining (Figure 1).

The main themes and sub-themes captured the strains and learning opportunities presented by the COVID-19 pandemic.

### 3.1. Theme 1: Burnout Manifestation

#### 3.1.1. Emotional Strain, Detachment and Irritability

The first sub-theme under burnout manifestation was the emotional strain, detachment and irritability attributable to the high number of critical patients, the number of deaths, feeling inadequate to help the sick, dealing with panicking patients and grieving patient relatives, as well as infected colleagues, and generally, the change in the nature of their practice. As a result of the high rate of mortality, resource constraints and concern for their own well-being, participants eventually developed some emotional detachment from their patients. This increased their irritability, thereby failing to react diplomatically in situations, and this irritability also affected family members in home settings.

*“it’s been hectic for all of us and most of us we’ve been hands-on, have been complaining majorly of the emotional strain that comes with it… It involved a lot of uhm- in fact long working hours and the high number of patients that we were seeing. So, it was involving mostly all of the aspects of our being. So, it came with emotional stress.”*

(D1)

*“You definitely experience the emotional exhaustion ‘cause you are losing patients. So that definitely takes a toll on you ‘cause [because] you feel like why am I even here if I can’t save the patients?... Patients are not the same, like, I mean, you’ll have a patient that will come in well, and then the next day patient has demised. Like that patient will affect me emotionally, more than someone who’s been unwell since the beginning.”*

(D2)

*“yeah It’s emotionally draining, it’s emotionally exhausting, because sometimes you would think I wonder what happened to the patient, because when I left, she was like this and this and this. And then when I come back… and that thing you would think about it for even a week.”*

(N4)

*“Uhm Because some of those patients were panicking it was emotionally exhausting, because we’d try to explain to someone uba (that) you have to do this and this and people were panicking, they’d take out the oxygen”*

(N3)

*“And it’s not just, it’s not just about the work itself, per se, during the second wave, it’s about the fact that you are not only dealing with the patient, you’re also dealing with their families, right. So, you are dealing with families, you’re also dealing with colleagues, colleagues with relatives that are also having severe form of COVID. So, there is a lot of emotional, I mean, you have- passing through a lot emotionally in terms of seeing your colleague losing their own relative, losing their own parent, losing your own colleague.”*

(D3)

Emotional strain also affected how HCWs cared for the patients, given that health outcomes were usually poor.

*“Because many times we know as health workers our aim is to salvage as much as possible of life as we can. But then during my COVID ward cover, many times, I had really little emotional attachment towards patients, and whatever the outcome I was not even moved. So, it was more like I’m just a robot doing work, I don’t really care much about the outcome because many times most of them will die. So, I think, personally, the main manifestation was my attitude towards patients, which is not yeah, ideal.”*

(D5)

The emotional strain and detachment had a ripple effect on HCWs, as they increasingly became irritable, even in circumstances where they would normally react diplomatically. This irritability affected family members in home settings as well.

*“A part of you empathizes and sympathizes with the patient right. But if- when there’s nothing else, you’ve given them salbutamol nebs and oxygen, sometimes they do not feel the oxygen it goes up to 15 up to 20 You feel like there’s nothing more I can do you feel like a bit irritated and tired as to, but there’s nothing else I can do, everything that was prescribed and above that I’ve done, but there’s nothing the patient is still in distress.”*

(N4)

*“So, you will, I think I had even myself personally noticed there was a time my wife said, “You know what, now you are, you no longer talk to the kids, you shout at them all the time… you get home you are tired physically, let alone emotional-emotional tiredness, you are physically tired. There’s emotion, there’s a child that does not understand what is going on. They just want their father’s love. So, you will, for a small thing, you will find shouting at the kids because you are tired... I was at that point of burnout.”*

(D6)

*“… and there are times you would even become irritable for no reason because we were going through a lot. There are times that you would find yourself not as receptive to the relatives of the sick one as much as you supposed to be and even towards colleagues because we would all be irritable at different points in time. So, it brought up characters we never thought we would be demonstrating to people around us.”*

(D1)

#### 3.1.2. Uncertainty-Induced Fear and Anxiety

The infectivity of COVID-19 and the mortality rate thereof induced fear and anxiety on HCWs. The fear of contracting and possibly dying from the disease while caring for patients was an overriding feeling, to a point of distrusting the personal protective equipment (PPE).

*“Mmh ok, I think a whole lot of us just had anxiety, like a lot of anxiety, ‘cause you’re doing so much but your patients are still dying so you feel like you’re not doing enough, but at the same time, we have our own anxiety with regards to- just like COVID, in the beginning anyway, with regards to COVID because you don’t know am I gonna get it? Did I sanitize correctly? Is my PPE—like did I wear my PPE correctly? Did I take it off correctly?... But I always was like thinking at the back of my mind, the smallest thing “I have a headache- do I have COVID?” “I have a scratchy throat- do I have COVID?” You know. So, I just walked around with a whole lot of anxiety so I didn’t actually actively do anything about it.”*

(D2)

*“You’re feeling unsafe for yourself and especially the people you live with at home, if you’re someone who lives with people, if it’s elderly people, if it’s kids. And every new wave brings a new characteristic or things like that. So, you’re always nervous you’re feeling unsafe, you’re unsure if you’re gonna make it that’s, I think that’s the biggest worry, unsure if ok I’m gonna work because it’s my job, but if I get it again, am I gonna be safe again?”*

(N1)

*“And patients are also anxious, the doctors are also anxious about what’s gonna happen, am I gonna get COVID and all that, yet you still have a lot of patients to see.”*

(D3)

#### 3.1.3. Physical Exhaustion, Yet, Low Job Accomplishment

HCWs also experienced physical exhaustion imposed by increased workload and work demands and long working hours; yet, there was no sense of job accomplishment, given their inability to save COVID-19-infected patients.

*“But then there’s also just like the physical fatigue ‘cause now you have so many more patients, even working in casualty uhm like you have so many more patients than you would normally, and your patients are so sick, so you like doing much more.”*

(D2)

*“And it was just feeling tired, working overtime. I remember some other days we would go out at 10 midnight because we had so much to do and the night staff would come late and there were few people to work around with, so personally it was very hard”*

(N2)

However, hard work did not match health outcomes, thereby creating a low sense of job accomplishment, and the fact that patients were indulging on information from the media platforms did not make things any better.

*“Now that patient will not have confidence in you… and now you will find yourself in the back foot in winning a battle with the patient. How is he gonna trust you about any other thing now that we’re gonna say. So, COVID will make you feel you are smaller when it comes to treating patients, especially if you treat someone who’s current with news and what is going on”*

(D6)

D2 added:

*“And the other thing about COVID, especially the first wave is that you sort of didn’t know what to do. So, you felt like you are powerless. It literally felt like you are powerless and… you were just standing there watching the patient die… I felt like I have no idea what I’m doing. And I’m just completely useless being here. Like, I felt like I’m just here for my patients to think, Okay, we have a doctor, but actually, this doctor doesn’t know what he’s doing, so yeah”*

N1 further attributed her inability to perform her duties efficiently to resource constraints.

*“You know what, if you have patients that are dying on your watch and you know there’s something you could’ve possibly done had the facility had such uhm treatment maybe, equipment maybe, that could’ve helped a patient at least, you go home feeling like a failure because you know “I studied this in the book, I could’ve done this if I had this to work with.”*

(N1)

D5 further expressed that not being able to achieve what you were called to do yields zero accomplishment.

*“I think the time I was covering COVID ward personally, the death rate, was more than 80%. So being a doctor, knowing that your call is to save lives, but then you’re not saving any. So, I don’t feel like I’m accomplishing anything, anything during the COVID Ward cover, because patients kept on dying, no matter what we tried to do. And so, it was like, zero accomplishment, just being there as a robot to certify deaths actually than to save lives.”*

#### 3.1.4. Dread and Professional Responsibility

The difficult period created a dreadful climate, took away HCWs’ enthusiasm about their work, but they still felt that they had no choice but to show up and fulfil their duties.

*“We cope, we push as much as… as hard as it was. But you had no choice, you wake up the following day you still come to work even though you know that it’s hard.”*

(N2)

*“And every time you have to wake up and come to work, it would be a struggle, because we were so exhausted. The interest in coming to work was almost zero. But because we had to push through, we didn’t have a choice we went on.”*

(D1)

### 3.2. Theme 2: Precursors of Burnout

#### 3.2.1. Occupational Exposure to High Mortality

Occupational exposure to high COVID-19 mortality rates imposed emotional exhaustion on HCWs in a manner that ultimately culminated in burnout.

*“So, because of the toll of seeing people dying every day, so initially, every time you get home, [you are] very emotionally exhausted. But then over time, like, I don’t really feel anything towards whatever’s happening to my patient… So the number one cause was the rate of death. So, there was a high mortality rate, and at the same time, not being able to do anything for the patients, there’s no medication, the hospital is poorly equipped. So, there’s nothing much we could do. So, the rate of death was so high that and at the same time not being able to intervene. So, I think that was the number one moral killer in a way… So, it was the first experience where by yes, you are a doctor and ahhh you have more deaths than people living. So, it was the first experience whereby, like, you see death left, right, and centre in a day, like that. So, I think it was- I had no previous encounters of such high death toll.”*

(D5)

*“And eh-eh during first wave and second wave there were lot of deaths as well, so that created a lot of emotional burnouts. It was not easy.”*

(N2)

#### 3.2.2. Staff Shortage

All participants conceded that staff shortage contributed to their feeling of burnout. The family medicine doctors were further stretched thinly because, even though they had their allocated time in the COVID-19 designated wards, they would still have to find the means to capacitate the casualty and outpatient departments.

*“Uhm there was one time that I was working with only one other category of nurse that was the lower category. And we had about 13 patients, just the two of us, 13 patients, majority of them were on oxygen and oxygen on respiratory distress, and others are very confused, trying to jump out of bed. “Nje” [So] it has been very hectic, it’s been made worse by a shortage of staff, because we do not have staff at all, at all. If you’re lucky, you’ll be like five in a team. And nurse-patient ratio is just not adding up, does not add up at all.”*

(N4)

Over the course of the pandemic, the hospital contracted nurses to meet the staff demands, and N2 shared how, during the times when there were no additional nurses, the burnout was more.

*“One may say that coz [because] in the beginning there was no one employed specifically for COVID, so we had to extract employees from the other wards to come and work in the COVID ward, so that created so much eh-eh-eh confusion and more of burnout, because people didn’t wanna [want to] come and work here, so we had short staff. We were so short staffed… So, imagine the staff that used to work in one ward having to be split into three wards and some of us are on quarantine. So, it was- there was that time though I’m not specifically sure was it wave 3 or wave 2. But I think it was wave 2, because it was most hectic of them all… But after we got our contract workers it became more easier as we got more staff to work around and uh-h things got better”*

(N2)

#### 3.2.3. Elongated High Patient and Workload Volumes

The COVID-19 era caused a rapid increase in patient volume in the hospital, thus increasing the workload and the number of hours for the personnel, particularly during waves 1 and 2 of the pandemic. COVID-19 exacerbated the distress experienced by HCWs prior to the pandemic.

*“The high number of patients that were coming were not easy to manage. So, we would get very large numbers, I would say more than 6 or 7 times the number of patients we would normally find before COVID or in between the waves. So, the major factors were the shortage of staff and the number of patients that were affected and needed that medical attention.”*

(D1)

*“You find that the workload now is too much on the people who are on the ground and the numbers would be quite high. So, I think during the second wave, it was just too much.”*

(D4)

#### 3.2.4. Disease Uncertainties

The rapid development and evolution of COVID-19 posed a certain level of uncertainty with regard to the knowledge of the disease, the required care and even the adequacy of personal safety precautions.

*“So, I think, during the first COVID wave, when- so obviously, none of us was really like, well up to date with COVID and how it works, except just seeing people dying a lot.”*

(D5)

*“So, it makes you feel like I don’t have- immediately feel like that’s what could easily have could have triggered people to go to burnout and even depression because who do you talk to, who knows better in this? There’s no one else. It’s you and what you read. Everybody is reading the same thing, it’s new every day. There’s something that is released. So, it makes you feel like you don’t- you’re isolated -you’re on your own. If you die, you will die and no one is sure how to treat- a definite treatment for the condition. It caused a lot of stress.”*

(D6)

Not knowing when one is protected enough from contracting the disease increased uncertainty, asserts N4:

*“No matter how much precaution you thought you took, the mask, the gowns, gloves, shoe covers, head covers, but there’s always that eish, maybe it’s I don’t know, because firstly, … when the disease was, was here first, it was said it was transmitted through droplets. So, we needed to wear mask. … now you kind of feel like, what if it’s airborne now? It’s no longer about droplets to need to be in a certain distance away from the person. What if it’s airborne now? So, a part of you feels like maybe the air can leak into the mask.”*

(N4)

#### 3.2.5. Consistent Feelings of Grief

The unfamiliar rapid disease progression left HCWs wondering about the constant experience of such catastrophic events, the deaths due to unclear pathologies. Dealing with grieving relatives left them with emotional strains beyond work environments, to a point of having dreams and nightmares, hearing the screams of relatives.

*“Because, at times, I would be sitting in my room thinking “how did we lose so and so?” or thinking; when I called a certain family, how a person reacted. Or maybe you would be sleeping, and then you wake up in a dream hearing a person crying. So, it does, like affect you as a person emotionally.”*

(N2)

*“No, no, no, definitely not. The- more than the patient dying in front of you, that has COVID, what-what-what, you-what would ehh remain something ringing in your mind is what the relative say, when they felt like as health professional, we didn’t do enough for their patients.”*

(D6)

### 3.3. Theme 3: Alleviating Factors of Burnout

#### 3.3.1. Time off Work

The doctors took rotational time off among themselves as a strategy to decrease exposure to COVID-19 infection, reduce burnout and also alleviate physical exhaustion.

*“Ok we also did like rotations, and we took weeks off so I think that also helps, ‘cause you’d come back from your week off think “ok I’m good, I can do this” Uhm and then you start the week and by Tuesday afternoon you “ok I need to go again”. So, it wasn’t always that bad, I think the breaks and the rotations made a difference.”*

(D2)

*“We tried to take time off, but it was not always possible. Sometimes you find that you, with the person that you’re working with, you may be in for two days, then the other one is off then when you come back, when the other one comes back. You take off just to relax. But it was not always possible that we will get off.”*

(D4)

#### 3.3.2. Psychological Intervention

HCWs also revealed that the psychological support they received from the hospital helped them cope better, and simply knowing that it is accessible was reassuring.

*“So, there’s so much emotion involved during the second wave. So that you felt that at the point that some of us will be needing counselling and the hospital actually provided avenue for those that felt that they needed counselling themselves, needed the support, they had that opportunity to get counselling, and so… I feel that the fact that you have that opportunity, that service available for you, the fact that you know that it’s available, so it’s already reassuring that okay, at a point, I may need to go there myself. Others went there, so, I definitely believe that it helped. The fact that it’s even available, is helping in that regard.”*

(D3)

*“I quickly tried to adjust the mind and it’s I think it’s also the time they tried to bring the psychologists to talk to us about the ways of coping and what the way how we can get help in getting our minds putting our minds at ease with the COVID. I think that played a crucial role, because it came in the first wave. So therefore, once we got there, and then you start to think, and then you come at peace, then you could cope unless you don’t have even that support-nyana [small support] from the psychology psychiatry side of things to be able to put things in patterns for you and do what you can.”*

(D6)

#### 3.3.3. Low Infection Rate Periods

The periods of low mortality and morbidity reduced burnout.

*“For-the- I think the fourth, third-fourth waves, the patients that we were seeing, what makes me feel like burnout was not as maximum as it was on the second wave, the number of patients that we were seeing was lesser and uhm the patients that we were seeing were not in very bad conditions. We would have the patients who are symptomatic, but quite stable, that wouldn’t need prolonged attention or mmh a long number of interventions to do. So, I think that’s what made us relax a bit, because the patients that we would be seeing were not in a severe disease, if I may put it that way, and the numbers were not many.”*

(D1)

#### 3.3.4. Additional Staff Members

The recruitment of additional nurses to meet the COVID demands meant that the workloads were fairly distributed, and the load became a little lighter.

*“But after we got our contract workers it became more easier as we got more staff to work around and uh-h things got better”*

(N2)

*“Oh, the contract and then was renewed at the end of January. So that how it was somewhat by remedial but it was only about two people that were added. So, it wasn’t that much of a help but it was not the same as only two people on duty with that number of patients.”*

(N4)

### 3.4. Theme 4: Every Cloud Has a Silver Lining

Even though the pandemic had been a very challenging time in the practice of these HCWs, causing them burnout, some good came out of it, in line with the saying “every cloud has a silver lining.” The COVID-19 pandemic brought some important lessons.

#### 3.4.1. Improved Infection Prevention Control Measures

The COVID-19 pandemic reinvigorated the basic infection prevention and control (IPC) measures, which had been neglected in the pre-COVID-19 era.

*“So, I think that’s one thing that it’s changed for me, it has made like, protecting myself so much more important, you know, now I work with “could this patient have TB?”, cannot really come close to him or her without wearing a mask. Like now it’s not just about COVID. And these are things that we should have been worried about even before. But we’re just very chilled about it… now I must always wear a mask when I come close to a patient, I must always wear gloves when I touch a patient, I must always sanitize like, before I touch a patient, after I touch the patient, like I literally go home at the end of the day, and my stethoscope is sticky, because after every patient I examined, I’ll clean it with the sanitizer.”*

(D2)

*“Uhm, I think more than anything, COVID has made us aware of any other things that we were not paying attention to like your cleanliness, your coughing and sneezing etiquettes and things like that. And now, I feel like we are trying, we are far from the end, but we are now trying to practice more of safer ways more cleaner ways, hygiene, and things like that. Ahh ja I feel like now we are more aware of such things than before, as practitioners and myself personally really.”*

(N1)

#### 3.4.2. Learning to Be More Empathetic Again

Some participants reported having returned to being empathetic again to their patients after COVID-19 caused emotional numbness.

*“So, it got us to be more receptive to our patients to be more empathetic to our patients. I think it’s- it’s out of, I don’t want to say it’s out of guilt for having not done it or for having been unable to do it during uhm the peak waves, but it has taught me specifically that, it has taught me how important it is to interact on an empathic level with the patients. It improves- it’s got eh-an impact on their healing on their way to healing, it’s got an impact on their way to disease resolution, as much you find that we attending to clinical conditions, but there is always an emotional and a psychological aspect of things. So, I feel like we are now more receptive to patients when it comes to their emotions and their psychological aspects in disease, specifically.”*

(D1)

*“Now a COVID patient doesn’t feel like just a patient with a diagnosis. Now, I want to understand cause my relative when he had COVID I mean, he was calling me all that he didn’t say he was scared because grownups, but like, I understood that, Ok, so as a doctor in the family, he feels like I’m someone that he can, someone that he can call and be asking questions about his condition and all that. So, I think I think now I’ve understood patients more. Yeah, or better, rather.”*

(D2)

#### 3.4.3. The Passion Remains and Confidence Grows

Some participants shared the zeal that they still have for the work they do, despite their burnout experience, while others recognized that the unusual COVID-19 experience presented an opportunity for learning new skills and growing their patient care experience.

*“Uhm, sheez..uhm, I feel like a soldier really (laughter). I feel like a soldier. I soldiered through a pandemic and contracting COVID three times and still coming to work and still not have quit my job. I feel like I am- I came out stronger. Obviously, there was burnout, there was things like that, but I feel like I came out strong. I came out more willing; want to know more, obviously do more with the employer’ support or the facility support because the most demotivating thing is wanting to do more and know more and not have the resources to do all that.”*

(N1)

*“So, in a way, because opened up my, my mind, professionally as to how to tackle and better intervene and better manage the patient. Because from past experiences as to how it was managed, so now you’re like, okay, this is how it was managed. Maybe if I try it like this, it might help.”*

(N4)

*“So, on my side when I see a severely ill patient I go with motivation because most of the management principles and the skills for managing these patients have improved a great deal. So, there is a lot of improvement of how we manage- on how I manage the patients that I see, especially now that we are in a relaxed environment. Taking from the experience that we would have to work under pressure and be in a hurry, so now that we are in less pressure it’s easier to be precise and it easier to be proper in managing our severely ill patients. I would say the positive part about it is that we developed, or we fine-tuned a lot of skills in managing these patients.”*

(D1)

*“But I can say now it has made me stronger, more independent, more eager but also more cautious of how I do things because anything can happen at any time.”*

(N1)

## 4. Discussion

The results of this study revealed how the COVID-19 pandemic rendered HCWs in MRH vulnerable to burnout, which manifested as emotional strain, physical exhaustion, anxiety and fear, low sense of job accomplishment, emotional detachment, irritability and dreading work, but without neglecting their responsibility. High COVID-19-related mortality, increased work volume, staff shortage and constant feelings of grief affected HCWs in COVID-19 wards. Psychological support from the hospital and the additional personnel mitigated the impact of COVID-19 on HCWs and improved their resilience.

Due to the COVID-19 morbidity and mortality, high workload, long working hours, resource constraints and feelings of job-related inadequacy related to poor recovery rates of patients, all participants experienced emotional strain. This was consistent with the literature, which posits emotional exhaustion as the central construct of burnout [3,34]. Similarly, a cross-sectional survey of 892 Spanish nurses working in COVID-19 hospital wards reported high levels of emotional fatigue, mainly associated with long working hours with COVID-19 patients and limited nursing experience [35]. However, in this study, we did not explore the relationship between work experience and burnout, but consistent with the literature, high workload, staff shortages and limited resources were important factors in burnout [3,11,24,36]. Similar to other studies [25] on COVID-19 and other viral outbreaks [34], we found that high mortality imposed emotional distress on HCWs. Poor health outcomes related to COVID-19 cases saw HCWs demonstrating negative attitudes towards the patients, developing callous feelings, in line with depersonalization, as one of three of Maslach’s dimensions of burnout [5,6]. Depersonalization is a way of coping with workplace-related emotional stress by guarding and moderating the level of compassion to patients [3]. This could also be called negative and callous attitudes towards patients [3,6]. In contrast to our results, nurses working in COVID-19 wards in Spain had low levels of depersonalization; instead, deeper connection with patients and empathy were formed [26]. However, in the literature, depersonalization among HCWs remains prevalent [11].

The impact of emotional strain and detachment led to irritability, which extended to home settings, a phenomenon congruent with the results of self-reported observation by Malaysian front-line HCWs who experienced burnout [37], frontline HCWs in Italy [38] and in Guillermo et al.’s meta-analysis [15].

COVID-19 created fear and anxiety among HCWs, a phenomenon not unique to this study [39], as they feared that they would contract COVID-19 and die or at least transmit the virus to their loved ones. This uncertainty left them anxious and fearful for what tomorrow held for them regarding their protection, to the point of distrusting the PPE at work [40]. This common occurrence of anxiety and fear among HCWs was not surprising, given the unpredictability and transmissibility of COVID-19 [18,25]. Molina-Mula et al. [35] also found that HCWs experienced anxiety and fear due to similar factors and also found the working experience a protective factor. HCWs were suspicious of any symptoms, such as headache or sore throat, especially if they had been around COVID-19-confirmed positive patients, as was the case in a study by Liu et al. [25].

Our study participants reported high levels of physical exhaustion, thereby threatening HCWs’ resilience at MRH. The high volumes of patients, increased job demands, long working hours in unfamiliar conditions and caring for critically ill patients with poor treatment outcomes were pervasive across the entire health system [18,24]. This was as strenuous an experience for HCWs as had been reported in China [24,25]. The extent of exhaustion was so severe that Liu et al. described their state as “collapsing” as soon as they reached their living quarters [25]. Physical exhaustion was also observed among the physicians in Jordan [18]. Sun et al. interviewed 20 nurses at First Affiliated Hospital of Henan University of Science and Technology who were caregivers to COVID-19 patients, and they described their physical exhaustion as extreme as well [32].

The fact that hard work put in by HCWs in caring for their patients did not match the outcomes created a feeling of inadequacy and powerlessness. Patients’ overindulgence in information from the media disempowered HCWs who did not have extensive knowledge of the disease. Their inability to save patients’ lives in their care created a sense of low accomplishment, which is one of the three dimensions of burnout discovered by Maslach and Freuderberger in the 1970s [1,5]. This is an important source of emotional distress for HCWs [24,41]. For them, there was no appropriate reward for their work, which is consistent with what has been found elsewhere [3,42]. De Hart also depicts five stages of burnout. Among them is the stage of chronic stress during which, he argues, individuals are made to feel like failures, ineffective in their roles and incompetent and inadequate [43]. Nurses in the United States of America also reported similar feelings during the pandemic [39].

The working conditions during the COVID-19 pandemic were so strenuous and unbearable, to the extent that HCWs dreaded going to work but held their heads above water to uphold their professional responsibility. This reluctance to work was also reported in a scoping review by Robertson et al. [9]. These results are similar to the findings of a Malaysian mixed-method study of over 1000 HCWs from various healthcare settings. Participants experienced a loss of enthusiasm for their work during the pandemic [37]. The South Korean study conducted during a Middle East Respiratory Syndrome (MERS) epidemic presented similar findings of HCWs opting to avoid working with infected patients but being bound by professional responsibility to provide care [44].

In our study, although not always possible, doctors made internal arrangements for rotational breaks to limit exposure to COVID-19 infection and reduce burnout and physical exhaustion. To meet the increased workload demands, which imposed a significant strain on HCWs, the government recruited additional nursing staff. These strategies are supported by the evidence, which suggests that long working hours are associated with poor inter-shift recovery, leading to more work stress and exhaustion, with the inverse being helpful in alleviating burnout during pandemics [23,36], and recruitment of either volunteers or part-time workers to give time-out to the permanent staff is recommended to reduce the strain [45] during the pandemic. This is in addition to adjusting staffing levels to meet the demands of the pandemic [46]. HCWs in China had the same need for more HCWs to meet the workload demands to ensure the quality of care [25].

Psychologist intervention was another important government intervention identified in our study. HCWs were provided with the option of psychological counselling sessions in order to equip them with pandemic coping and protective strategies. The knowledge of the availability of this option was soothing for HCWs, a strategy that had been used in China [25] and in other previous viral outbreaks [18,23]. This strategy can also be used to minimize risk for those on the pandemic frontline but not yet experiencing burnout [23,40].

On the brighter side, and without downplaying the devastations of the COVID-19 pandemic, participants reported high awareness of IPC measures, such as screening patients, maintaining hand and respiratory hygiene, and wearing masks and PPE, because of COVID-19. These IPC measures had been neglected in the pre-COVID-19 era, negating the WHO recommendations on protection measures against viral outbreaks [47,48]. Consistent with these reports, HCWs in China also reported improved IPC compliance during the COVID-19 outbreak [47], and strict IPC guidance was reported to alleviate stress [49].

Despite undergoing an experience of emotional detachment towards their patients, the participants in our study eventually regained empathy and compassion, as COVID-19 affected their close relatives. This shift compelled HCWs to recognize the suffering of their patients and understand that they are treating the person and not the illness, noting that empathy goes with listening, respecting, communicating well and understanding. These are the backbone of effective medical care [19,50].

Qualitative studies are important in order to complement quantitative studies and create a holistic picture of all the challenges underpinning burnout, especially within the context of COVID-19. As a strength of this study, the rigorous method of data analysis employed illuminates the deeper level of HCWs’ shared real-life experiences of burnout. To our knowledge, there are no COVID-19-related burnout studies on HCWs in the Eastern Cape Province, and contrary to other studies, our study showed that despite the dark cloud imposed by COVID-19, there is a silver lining.

Although the study produced important findings, especially for MRH management, the transferability of the findings would be limited, given that the study was conducted in one health facility and confined to nurses and doctors, thereby excluding other healthcare workers involved in the care of COVID-19 patients. Although we contend that data saturation was reached, additional insights may have emerged with a larger sample size. Secondly, the study did not make comparisons between the two professions (nurses and doctors), as that fell outside the study objectives. Future studies should consider incorporating other healthcare professionals involved in patient care using mixed methods.

## 5. Conclusions

Healthcare workers are the backbone of effective healthcare services, but the nature of their work predisposes them to burnout. The COVID-19 pandemic increased HCWs’ susceptibility to burnout, given that they had to work under highly unbearable conditions, where the job demands increased unexpectedly and rapidly. In this study, HCWs experienced various burnout symptoms, including emotional strain and detachment, uncertainty-induced fear and anxiety, physical exhaustion, low job accomplishment and dreading work while maintaining professional responsibility. The conditions created by COVID-19 pushed the government and HCWs alike to think outside the box and be creative in mitigating COVID-19-induced burnout. These creative ways included rotational time off work and psychological support.

Most importantly, although the COVID-19 conditions initially threatened HCWs’ resilience, this subsequently improved HCWs and their adherence to IPC. The burnout phenomenon, which has evolved over the years in terms of what it entails and its evocative power to capture the realities of people’s workplace experiences, has made it both important and controversial in the research field [3]. Outbreaks such as COVID-19 magnify the depth and magnitude of the phenomena, and therefore, more studies capturing the various experiences of HCWs and patient perspectives, using mixed methods, are essential. This study provides strategic information for policymakers and managers on developing and strengthening welfare policies to promote and protect frontline HCWs’ well-being and work functioning.

## Data Availability

The datasets analyzed during the current study are the property of the University of KwaZulu-Natal (UKZN) and cannot be publicly available. All interested readers can access the dataset from the UKZN Biomedical Research Ethics Committee (BREC) through the following contacts: The Chairperson Biomedical Research Ethics Administration Research Office, Westville Campus, Govan Mbeki Building University of KwaZulu-Natal P/Bag X54001, Durban, 4000 KwaZulu-Natal, South Africa Tel.: +27-31-260-4769 Fax: +27-31-260-4609 Email: BREC@ukzn.ac.za.

## Abbreviations

HCW	Healthcare worker
MRH	Mthatha Regional Hospital
SSA	Sub-Saharan Africa
COVID-19	Coronavirus Disease 2019
MBI	Maslach Burnout Inventory
PPE	Personal protective equipment
IPC	Infection prevention and control
MERS	Middle East Respiratory Syndrome
WHO	World Health Organization
TB	Tuberculosis
UKZN	University of KwaZulu- Natal
BREC	Biomedical Research Ethics Committee
CIDERU	Cancer & Infectious Diseases Epidemiology Research Unit (CIDERU)
